# A Novel Oxidative Stress-Related lncRNA Signature That Predicts the Prognosis and Tumor Immune Microenvironment of Breast Cancer

**DOI:** 10.1155/2022/9766954

**Published:** 2022-10-12

**Authors:** Jinlai Zhao, Haiyan Ma, Ruigang Feng, Dan Li, Bowen Liu, YueYu, Xuchen Cao, Xin Wang

**Affiliations:** ^1^The First Department of Breast Cancer, Tianjin Medical University Cancer Institute & Hospital, National Clinical Research Center for Cancer, Tianjin 300060, China; ^2^Tianjin's Clinical Research Center for Cancer, Tianjin 300060, China; ^3^Key Laboratory of Breast Cancer Prevention and Therapy, Tianjin Medical University, Ministry of Education, Tianjin 300060, China; ^4^Key Laboratory of Cancer Prevention and Therapy, Tianjin 300060, China; ^5^Department of Gastrointestinal Surgery, Central Hospital of Tangshan, Tangshan, Hebei 063000, China; ^6^Department of General Surgery, Second Central Hospital of Baoding, Baoding, Hebei 071000, China

## Abstract

**Background:**

The association between oxidative stress and lncRNAs within the cancer-related researching field has been a controversial subject. At present, the exact function of oxidative stress as well as lncRNAs exert in breast cancer (BC) are still unclear. Therefore, the present study examined the lncRNAs oxidative stress-related in BC.

**Methods:**

Transcriptome data of BC obtained from TCGA (The Cancer Genome Atlas) database were used to generate synthetic matrices. Patients with breast cancer were randomly assigned to training, testing, or combined groups. The prognostic signature of oxidative stress was created using the selection operator Cox regression method, and the difference in prognosis between groups was examined using Kaplan-Meier curves, the accuracy of which was calculated using a receiver-operating characteristic-area through the curve (ROC-AUC) analysis with internal validation. Also, the Gene Set Enrichment Analyses (GSEA) was applied for the analysis of the risk groups. To conclude, the half-maximal inhibitory concentration (IC50) of these groups were investigated by immunoassay assay.

**Results:**

A model based on 7 lncRNAs related to oxidative stress was proposed, and the calibration plots and projected prognosis matched well. For prognosis at 5, 3, and 1 year, the area under the ROC curve (AUC) values were 0.777, 0.777, and 0.759. The functions of target genes identified by GSEA appear to be mainly expressed in metabolism, signal transduction, tumorigenesis, and also the progression. The remarkable differences in IC50 and gene expression between risk groups in this study provide a deep insight for further systemic treatment. Higher macrophage scores were acquired in the high-risk group, of which patients showed more response to conventional chemotherapy drugs, such as AKT inhibitor VIII and Lapatinib, as well as immunotherapy strategies including anti-CD80, TNF SF4, CD276, and NRP1.

**Conclusion:**

The prognosis of breast cancer can be independently predicted by the markers, which sheds light on further research of the specific role of lncRNAs which are oxidative stress-related and clinical treatment of breast cancer.

## 1. Introduction

Breast cancer is the most commonly diagnosed feminine malignant tumor with an increasing incidence. Studies have shown that in 2018 there were approximately 2.08 million new diagnosed cases and 630,000 deaths globally [1]. With the improvement of surgery, radiotherapy, and chemotherapy, the overall survival condition of breast cancer patients has improved significantly. However, breast cancer is insidious in its onset and highly malignant. By the time a patient presents with typical symptoms of BC, the tumor has often progressed to an intermediate to advanced stage. At this time, common interventions are less likely to yield desired results, and this significantly affects the prognosis of patients [2, 3]. Therefore, it is very important to dig into the molecular biological mechanism of breast cancer and to find molecular biological markers for early identification and development of breast cancer.

Oxidative stress refers to the overproduction of highly reactive molecules including reactive oxygen species (ROS) and reactive nitrogen species (RNS), by the body in response to various damaging stimuli. Physiological and pathological reactions in cells and tissues are caused by the imbalance of oxidation-antioxidation *in vivo*. Many factors, such as radiation, age, infectious diseases, and heat stress, may lead to increased intracellular ROS concentrations, which stimulate intracellular oxidative stress response and protect or destroy cells [4]. In recent years, scholars have gradually clarified the participation of oxidative stress in the occurrence and prognosis of tumors. Oxidative stress causes DNA mutations in tumor cells, mediates the action of proto-oncogenes, and causes aberrant cell amplification and tumor formation [5]. Moreover, oxidative stress can also promote the metabolism of tumor by altering the key enzymes of metabolism, inducing changes in the metabolic genome and activating signaling pathways, thus promoting the further development of tumors [6].

Long noncoding RNA (lncRNA) is considered as one of the important members among the noncoding RNA family, whose length was more than 200 nucleotides and are a subtype of RNA transcripts [7]. In recent years, researches have proved that multiple lncRNAs participate in various biological processes as a vital part, especially in the incidence and progression of invasive tumors. Many lncRNAs have been illustrated to be closely linked to the breast cancer development and can be broadly classified into two types: cancer-promoting and cancer-inhibiting [7]. Their mechanisms of action are to affect the amplification, invasion, distant metastasis, apoptosis, and drug resistance of breast cancer cells. lncRNAs which are oxidative stress-related have not been studied in breast cancer.

In this research, we aimed to identify lncRNAs associated with oxidative stress regarding breast cancer and to elucidate the participation of lncRNAs in tumor microenvironment (TME) and breast cancer prognosis. To identify the underlying mechanisms, a gene enrichment analysis was performed.

## 2. Materials and Methods

### 2.1. Data Identification and Acquisition of Oxidative Stress-Associated lncRNAs

In order to obtain comprehensive data matrices about BC with normal tissue, the RNA transcriptome datasets (HTSeq-FPKM) and the germane clinical information were acquired online from The Cancer Genome Atlas (TCGA) database (https://http://portal.gdc.cancer.gov/). 1109 BC tissue samples and 113 normal breast tissue samples were acquired as control samples. Furthermore, extensive clinical information on patients was obtained from the TCGA. Samples with a follow-up period of less than a month were excluded from further screening using clinical information. As all the data enrolled were obtained from The Cancer Genome Atlas Database and strictly followed guidelines of TCGA publication (http://cancergenome.nih.gov/abouttcga/policies/publicationguidelines), ethics committee approval was not required.

### 2.2. Screening Analysis of Oxidative Stress-Related Genes and lncRNAs

The lncRNA profiles were acquired firstly from the dataset of RNA seq. Total RNA expressing panel was normalized before the analyzation through log2 transformation. A list of genes related to oxidative stress was downloaded from an online website (https://www.genecards.org/) to screen for gene sets associated with oxidative stress with a correlation score greater than 7.

### 2.3. Functional Enrichment Analyzation of Differentially Expressed Genes Linked to Oxidative Stress

**Figure 1:**
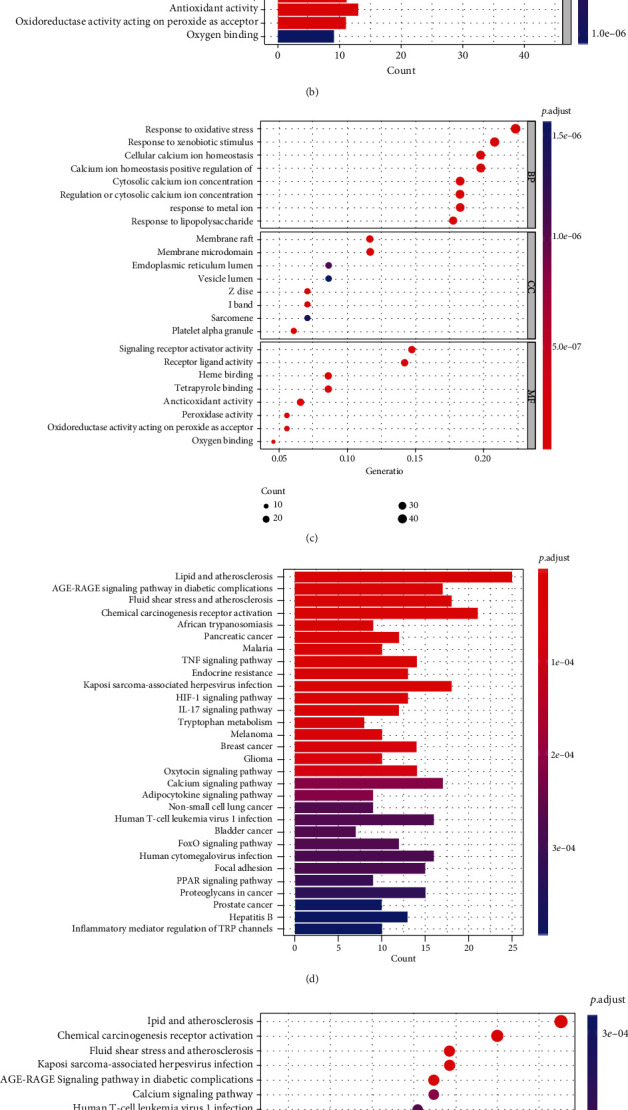
GO and KEGG analyzing of DEGs related to oxidative stress in cancer and normal tissues. (a) Volcano plot of 794 genes related to oxidative stress in BC. Light salmon dots represent for upregulated genes and blue dots for downregulated ones. (b, c) GO analysis of DEGs related to oxidative stress. (d, e) KEGG analysis of DEGs related to oxidative stress. GO: Gene Ontology; DEGs: differentially expressed genes; KEGG: Kyoto Encyclopedia of Genes and Genomes; fdr: false discovery rate; FC: fold change; CC: cellular components; BP: biological process; MF: molecular function.

A false discovery rate (FDR) < 0.05 and |log2 − fold change (FC) > 1| were applied in this experiment as screening criteria to acquire the panel of oxidative stress-related different expressing genes (DEGs). Gene Ontology (GO) were conducted for the research aim, as well as Genes Kyoto Encyclopedia and Genomes (KEGG) analyzation in the “ggplot2” package (Figure 1).

### 2.4. Identification of Prognostic lncRNAs Related to Oxidative Stress

We utilized the “limma” package for the calculation of the correlation between genes related to oxidative stress as well as lncRNAs. The square of correlation coefficient |*R*^2^| > 0.3 in combination of *p* < 0.001 was identified as lncRNAs which are oxidative stress-related. We performed univariate Cox regression analysis for lncRNAs which are oxidative stress-related associated to the cancer prognosis in breast cancer patients, followed by Lasso Cox regression and multivariate Cox regression analyzation of lncRNAs which are oxidative stress-related for constructing the predictive signature of lncRNAs which are oxidative stress-related. The computational equation adapted is descried as follows:

(1) $$\begin{array}{c} \text{risk score}=\overset{n}{\underset{i=1}{\sum }}*\left({\text{Coef}}_{i}*x_{i}\right). \end{array}$$

Coef stands for the coefficient value, and *x* for selected lncRNAs expressing value. This formula was utilized to assess the risk score for each individual diagnosed with breast cancer. The patients were divided into two separate groups on the basis of the median risk score: low-risk along with high-risk groups [8, 9]. Differences of survival condition between groups were compared through the log-rank test.

### 2.5. The Prognostic Model Development

A model for independent prognostic was developed using Cox regression. Nomogram was applied for the prediction of the patient survival. The calibration curves, receiver-operating characteristic (ROC), and concordance index (*C*-index) curves were developed for exploring this model's accuracy. Demographic variables were included in the multivariate Cox regression analysis to see if the risk score could independently predict the development of breast cancer. The stability of the prediction model conducted in this experiment was also examined within the testing and training groups.

### 2.6. Functional Analysis

The online CBioPortal (http://www.cbioportal.org/) was taken to describe the mutation profiles of each key gene. Gene set enrichment analysis was applied for interpreting the functional enrichment of gene expressing panel [10]. The enrichment of lncRNAs related to oxidative stress with a classified prognosis value was explored and 10 GO and KEGG pathways related to oxidative stress were visualized.

### 2.7. The Investigation of the Immunocheckpoints, TME, and the Model in the Clinical Treatment

Limma, GSVA, ggpubr R, and ggplot2 packages as well as GSEABase were utilized to determine the expression differences of 29 immunocells and 47 immune checkpoint genes within the studied groups and to guide the immunotherapy of breast cancer [9]. “pRRophetic,” “ggpubr,” “ggplot2,” etc. R packages were applied to classify the differential expression of IC50 in the two groups of breast cancer and to perform clinical chemotherapy against breast cancer [11].

### 2.8. Statistical Analysis

All statistical analyzation involved were completed using R software (Version 4.1.2). The Wilcoxon test was used to compare the expression levels of DEGs in cancer and normal tissue samples. Univariate Cox regression analyzation was performed for determination of the relationship of lncRNAs which are oxidative stress-related with overall survival, and lncRNAs which are oxidative stress-related were screened using multivariate Cox analysis for the construction of predicting signature discussed in this research. The Kaplan-Meier method combined with log-rank test were applied for analyzation of the OS of patients in the two groups. The “survival ROC” package was applied for drawing the ROC curves and for determination of the area below the curve (AUC) values. Principal component analysis (PCA) method was utilized to discover the distribution of patients ranked at differed risk scores. Statistical tests turned out to be bilateral, with *p* < 0.05 being significant.

## 3. Results

### 3.1. Identification of Prognostic lncRNAs Which Were Oxidative Stress-Related

**Figure 2:**
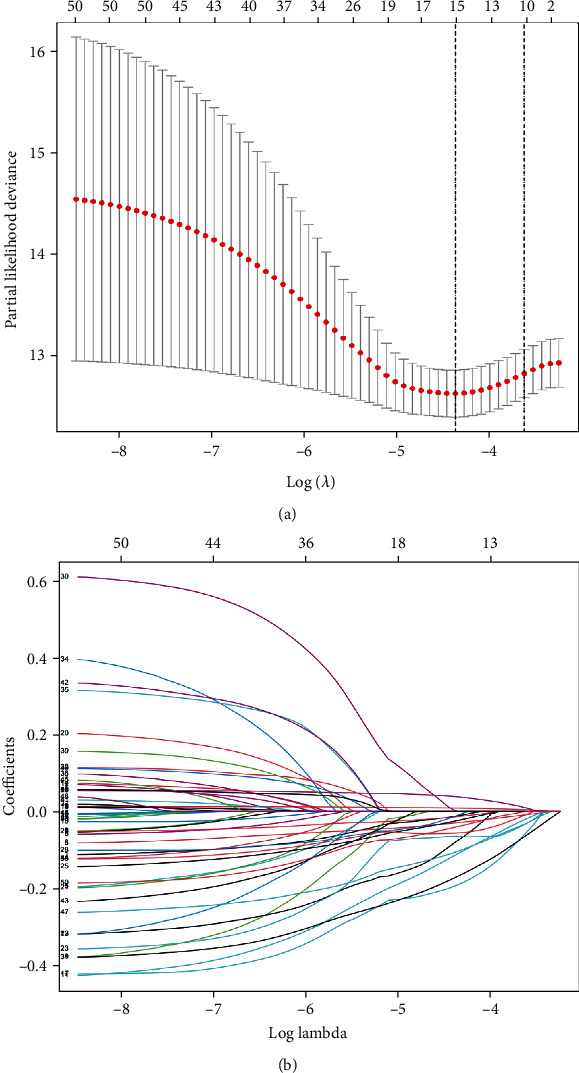
Oxidative stress-associated lncRNA selection through the screening of Lasso model. (a) Lasso coefficients of the 15 lncRNAs which are oxidative stress-related in BC, where the optimal log (lambda) value is marked by vertical dashed lines. (b) Lasso coefficient profiles.

**Figure 3:**
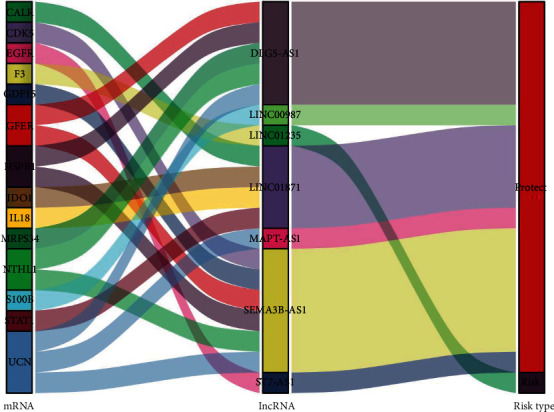
The Sankey diagram of prognostic.

14,142 counted lncRNAs were gained from TCGA-COAD, among which 1086 lncRNAs linked to oxidative stress were identified. Univariate Cox regression analyzation uncovered that 50 of them were linked to the development of BC. Lasso Cox regression analyzation displayed in Figure 2 showed that 15 lncRNAs which are oxidative stress-related had a connection with the BC development. Finally, multivariate Cox regression analysis uncovered that 7 lnccRNAs which are oxidative stress-related were linked to the development of BC. DLG5-AS1, LINC01235, SEMA3B-AS1, LINC00987, ST7-AS1, MAPT-AS1, and LINC01871 were identified as construct predictive signatures (Table 1). The risk scores were calculated as: risk score = − 0.65107016 × DLG 5− AS 1 expressing level + 0.40027496 × LINC 01235  expressing level + − 0.244710708 × SEMA 3 B − AS 1 expressing level + − 0.684301493 × LINC 00987 expressing level + − 1.181200718 × ST 7 − AS 1 expressing level + − 0.781949407 × MAPT − AS 1  expressing level + − 0.713957256 × LINC 01871 level. The lncRNAs were further visualized with the ggalluvial, ggplot R software package. From the Sankey diagram, one lncRNA (LINC01235) was a detrimental prognostic factor, and the others (DLG5-AS1, SEMA3B-AS1, LINC00987, ST7-AS1, MAPT-AS1, and LINC01871) were positive prognostic factors (Figure 3).

### 3.2. The Prognostic Impact of the Signature Established

**Figure 4:**
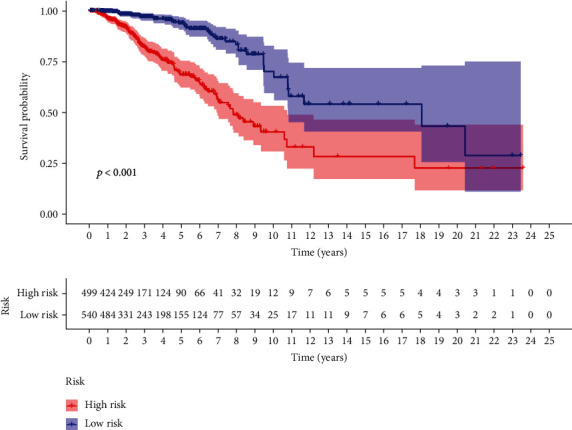
The KM survival curve of risking score on the basis of 7 lncRNAs which are oxidative stress-related.

**Figure 5:**
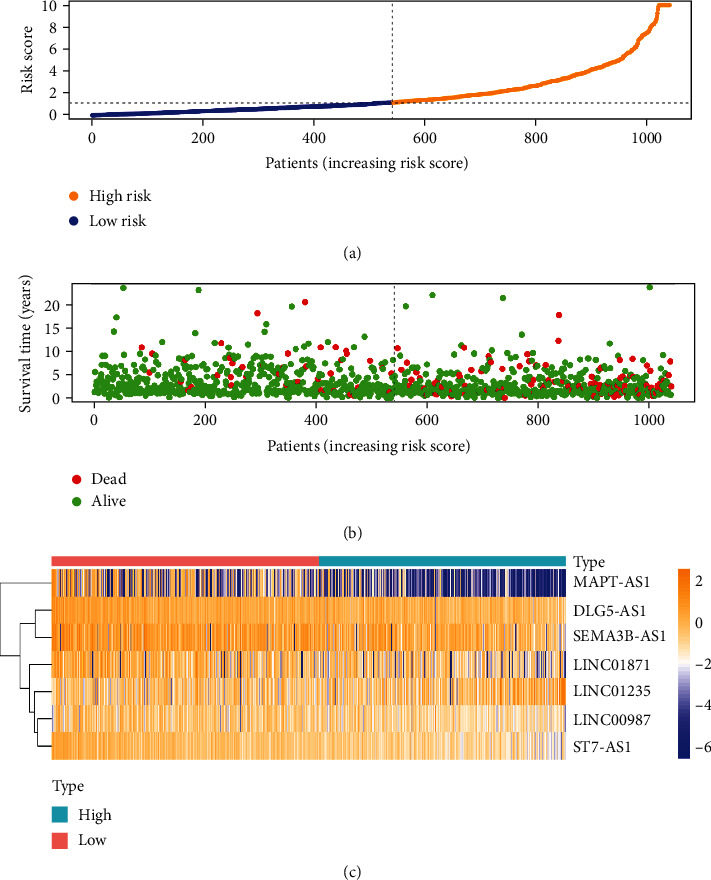
The analysis of signature regarding lncRNA which are oxidative stress-related for BC patients. (a) The risk scores of the investigated groups. (b) The patients' survival time. (c) Heat map of the ten lncRNA expression. As the color shifts yellow, the expression level becomes higher.

Risk score was linked to the survival condition of BC patients significantly. There was a shorter OS in the group with high-risk (*p* < 0.001, log-rank test) (Figure 4). Cox regression suggested significant developing impact on the risk score for the BC patients (Figure 5).

### 3.3. Clinical Value of the Signature regarding lncRNA Oxidative Stress-Related

**Figure 6:**
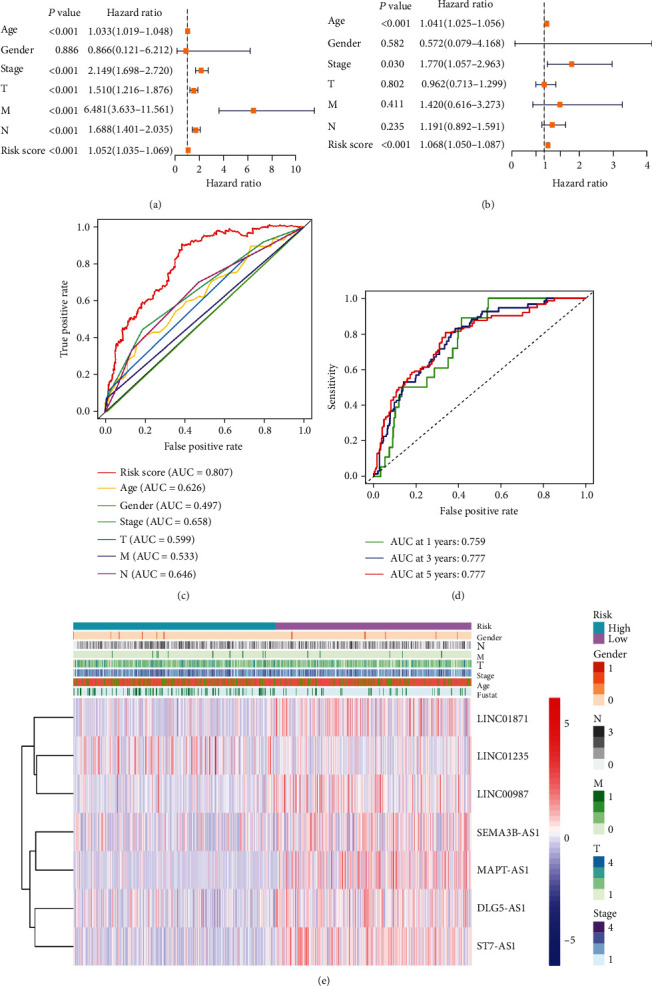
The correlation of the predictive signature with the development of BC. (a) The forest plot regarding univariate Cox regression analyzation. (b) The forest plot regarding multivariate Cox regression analyzation. (c) The ROC curve illustrating the clinicopathological variables and the risk scores. (d) ROC curves along with corresponding AUCs at 1-, 3-, and 5-year survival with the predictive signature. (e) The heat map of distribution for the clinicopathological variables and seven prognostic lncRNAs in the two risk groups.

The results of univariate cox regression analysis suggested that general information including age, T stage, N stage, M stage, stage, and risking score was related to the survival condition in BC patients (Figure 6(a)). As suggested by multivariate Cox regression analyzation, age and risk score appear to be separate predictors of OS in BC patients (Figure 6(b) and Table 2). The AUC of the risk score was 0.807, which outperformed clinicopathological variables in predicting the development of BC (Figure 6(c)). The AUCs of 5-, 3-, and 1-year survival ratios were accordingly recorded as 0.777, 0.777, and 0.759, which indicated positive predictive capability (Figure 6(d)). The clinicopathological variable differences between the groups were analyzed, while N stage (*p* < 0.05) along with stage (*p* < 0.05) were uncovered to be different between the two groups discussed (Figure 6(e) and Table 3).

**Figure 7:**
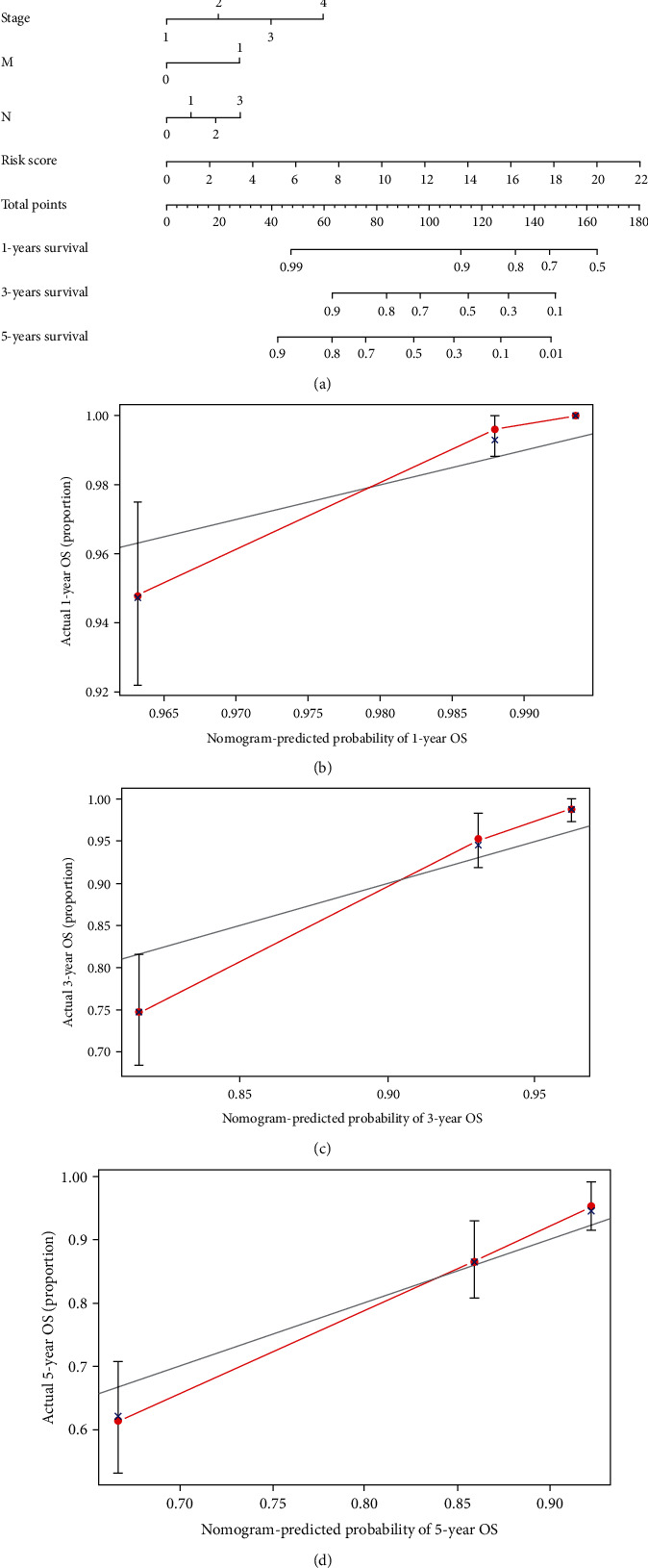
The nomogram construction and verification. (a) A nomogram in combination of risk scores and clinicopathological variables. (b–d) Calibration curves across the actual and predicted OS rates at 1, 3, and 5 years.

To predict the development of breast cancer further, a nomogram including clinicopathological variable as well as the risk score was constructed, which could predict the 1-, 3-, and 5-year prognosis (Figure 7(a)). Curves of calibration implied a positive consistency of the actual OS conditions along with the predicted survival conditions at separate period (Figures 7(b)–7(d)).

### 3.4. Internal Validation of the Predictive Characteristics

**Figure 8:**
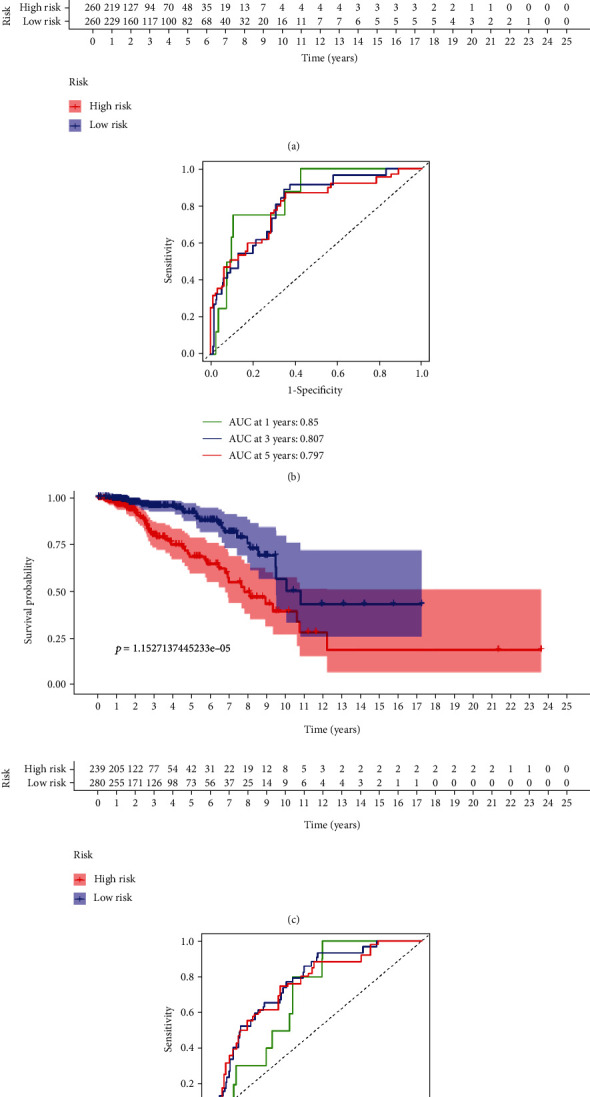
Internal confirmation of the predictive feature for over survival condition on the basis of the TCGA dataset. (a) Kaplan-Meier survival curving plot in the internal training cohort. (b) ROC curving plot and AUCs at 1-year, 3-year, and 5-year survival condition in the training internal cohort. (c) Kaplan-Meier survival curving plot in the internal testing cohort. (d) ROC curving plot and AUCs at 1-, 3-year, and 5-year survival in the internal testing cohort. AUC: area under the curve; ROC: receiver-operating characteristic; TCGA: The Cancer Genome Atlas; OS: overall survival.

For the verification of the applicability of the predictive characteristics for OS on the basis of the TCGA dataset, 856 patients with BC were randomly separated into two grouping cohorts (training cohort *n* = 427, test cohort *n* = 429). The demographic information of patients enrolled are illustrated in Table 4. Complying with the results observed, OS rates of patients in the group with high-risk tended to be lower (*p* = 9.65*e* − 10). (Figure 8(a)) In the testing cohort, the prognosis of the group with high-risk turns to be worse (*p* = 1.15*e* − 05) (Figure 8(c)) The ROC curves of two cohorts appears to be a positive predictive capability. In the training cohort, the AUCs of 5-, 3-, and 1-year prognosis conditions were, respectively, 0.797, 0.807, and 0.85 (Figure 8(b)), while within the test cohort, the AUCs of 5-, 3-, and 1-year survival conditions were, respectively, 0.747, 0.761, and 0.689. (Figure 8(d)).

### 3.5. Function Analyzation

**Figure 9:**
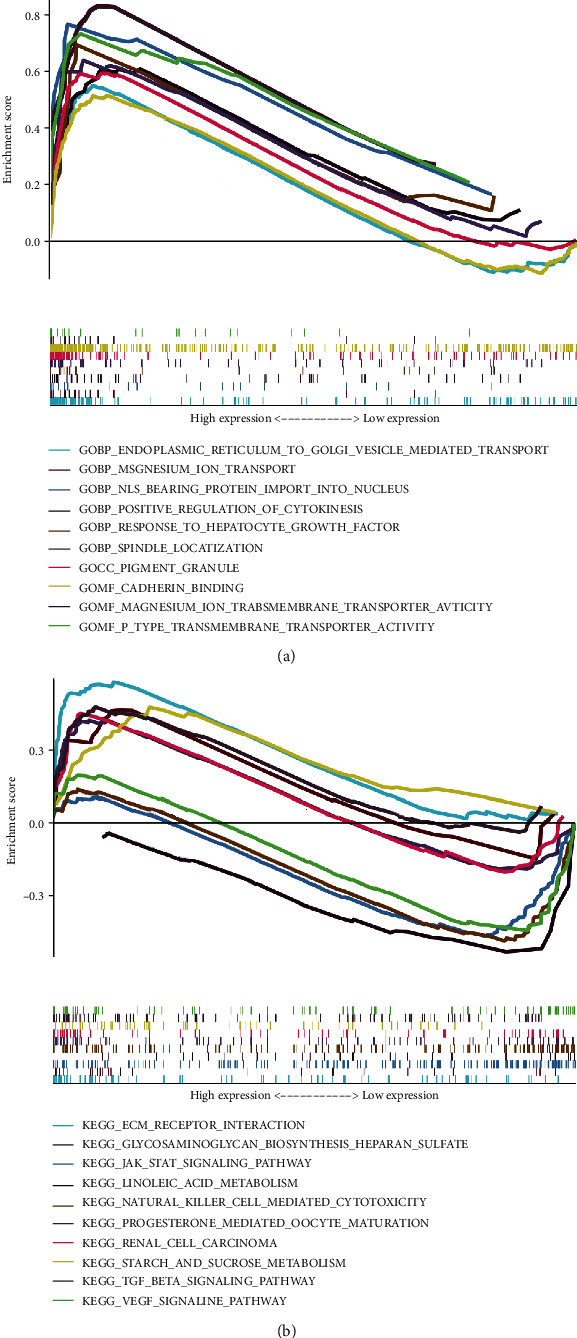
The functional analysis based on lncRNAs which are oxidative stress-related. (a) GO; (b) KEGG.

5484 GO analysis and 178 KEGG analysis were conducted. In GO analysis, the lncRNAs oxidative stress-related were enriched in biological processes like regulation of cell cycle and of mitosis (Figure 9(a)). KEGG analysis uncovered that these lncRNAs were mainly enriched into metabolism, malignant tumor formation, signal transduction, etc. (Figure 9(b)). Furthermore, it was proposed that the gene clusters were associated to critical biological processes, genesis functional pathways, and cancer prognosis, for example, JAK-STAT as well as VEGF signaling pathway (*p* < 0.05) was solidly linked to the cancer invasion and metastasis.

### 3.6. Immune Cell Infiltration

**Figure 10:**
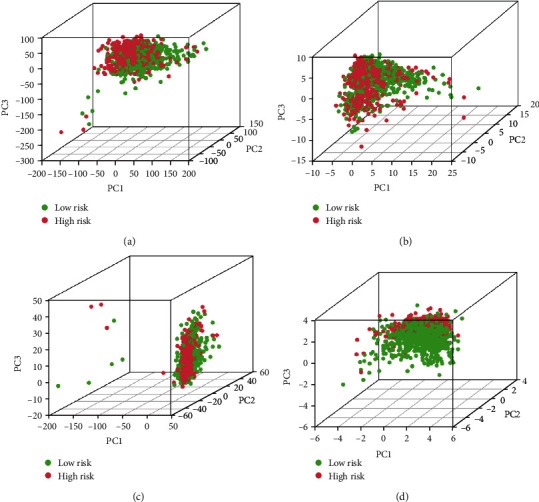
Patients with different risk scores have different oxidative stress statuses. PCA maps show the distribution of patients on the basis of the (a) complete genome; (b) oxidative stress-related gene clusters; (c) lncRNAs related to oxidative stress; and (d) lncRNA feature. The segmentation across the red and green dots becomes stronger when tested using only signature lncRNA.

With PCA maps, it was feasible to visualize the patients' distribution based on oxidative stress-related gene sets, the entire genome, oxidative stress-related lncRNAs, and important genes. The results implied that the key gene appears to be the best for patients. Patients with differential risking score were distributed in differed quadrants (Figure 10).

**Figure 11:**
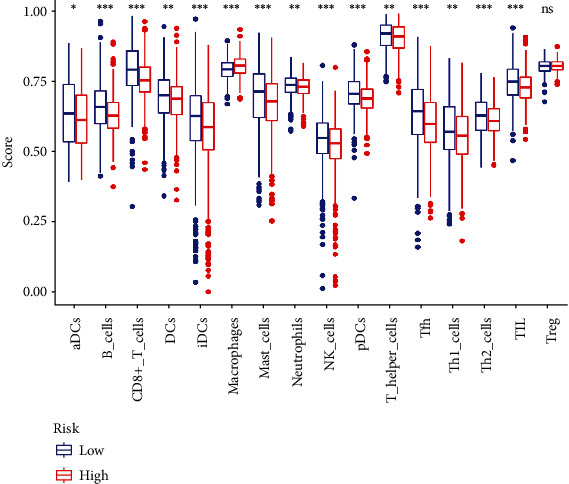
The immune infiltrating cell scores in high-risk as well as low-risk groups.

To discover the correlation between risking score and immune cells further, the GSEA enrichment scores for different immune cell clusters were assessed. The results showed DCs, aDCs, B_cells, plasmacytoid dendritic cells (pDCs), CD8+_T_cells, mast cells, immature dendritic cells (iDCs), neutrophils, macrophages, NK cells, T follicular helper (Tfh) cells, tumor infiltrating lymphocyte (TIL), T helper cells, T helper type 1 (Th1) cells, and T helper type 2 (Th2) cells were significantly varied between the groups discussed (Figure 11). Only macrophages in the group with high risk exhibited a high score, suggesting that the function of macrophages was more active.

### 3.7. Linage Between the Predictive Signature and BC Therapy

**Figure 12:**
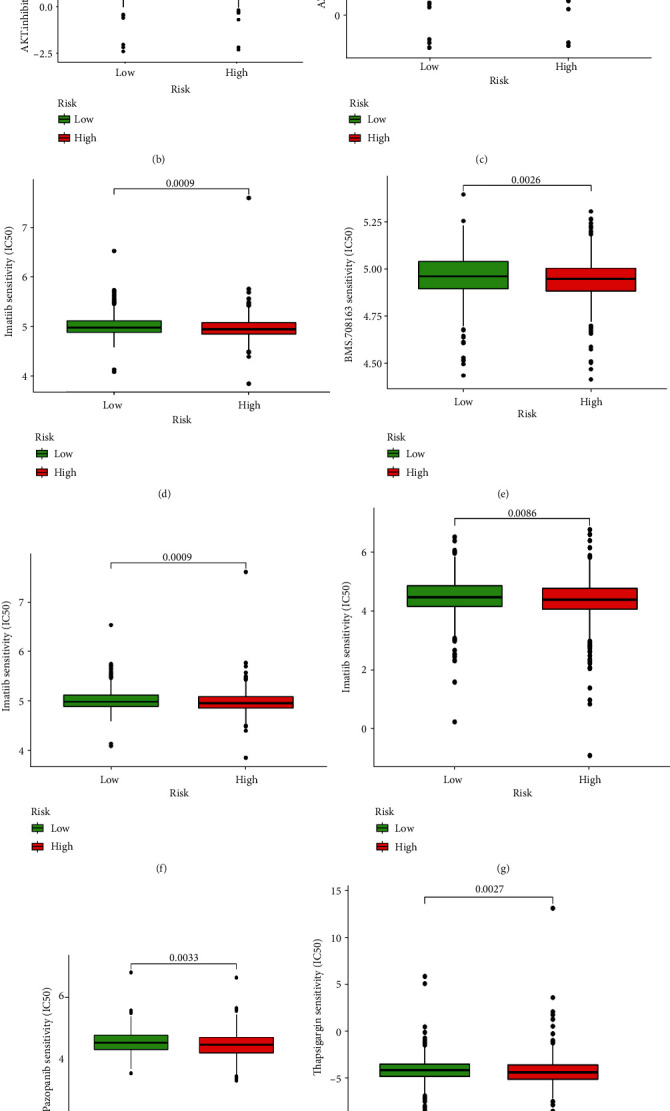
Comparison of sensitivity to treating drugs across high- and low-risk groups. (a) CD80, TNFSF4, CD276, and NRP1 expressions between groups. (b) IC50 of AKT inhibitor VIII between groups. (c) IC50 of AZD6482 between groups. (d) IC50 of bicalutamide between groups. (e) IC50 of BMS.708163 in the two risk groups. (f) IC50 of imatinib in the two risk groups. (g) IC50 of lapatinib in the two risk groups. (h) IC50 of pazopanib between groups, (i) IC50 of thapsigargin in the two risk groups, and (j) IC50 of methotrexate between groups.

The expression of CD80, TNFSF4, CD276, and NRP1 was higher significantly in the group with high risk, suggesting a potential response to anti-CD80, TNFSF4, CD276, and NRP1 immunotherapy in high-risk patients (Figure 12(a)). This provides a new therapeutic target for immunotherapy of BC. Combined with immunotherapy, we also surveyed the linkage between the predicting feature and the general chemotherapy efficacy, then revealed that the AKT inhibitor VIII, AZD6482, bicalutamide, BMS.708163, imatinib, lapatinib, pazopanib, and thapsigargin in the high-risk group exhibited a lower IC50 compared with the other group (Figures 12(b)–12(i)), and the methotrexate exhibited a higher IC50 in the group with high-risk (Figure 12(j)), which could help explore personalized treatment schemes appropriate for both high- and low-risk group individualized patients.

### 3.8. Mutation Landscape of Key Genes

**Figure 13:**
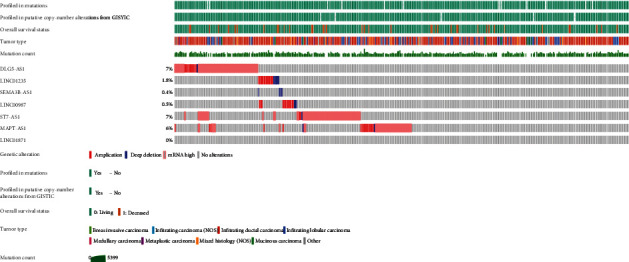
Mutations in the seven key genes, based on BC data in TCGA. Bar plots showing mutations in the seven key genes.

The OncoPrint view of key genes in the CBioPortal database were applied to visualize mutations within the seven key genes on the basis of data acquired from 1084 BC patients. Nearly 1/4 of these patients (23.7%) had mutations in all seven key genes. The highest rate of mutations was found in DLG5-AS1 (7%) and ST7-AS1 (7%) (Figure 13).

## 4. Discussion

Breast cancer looks to be a large malignant tumor that endangers both women's physical and mental well-being. The incidence of BC appears to be growing year by year in recent years, with a definite trend toward younger age. It is therefore essential to establish an accurate tool for the prediction of the development of BC to guide clinical diagnosis and treating strategy.

Tumor generation is a complex multistep process requiring three stages: onset, promotion, and development. A large number of studies have illustrated that reactive oxygen species (ROS), products of oxidative stress, are involved in all stages of tumor formation [12]. Tumorigenesis is closely correlated with ROS-induced oxidative damage to nuclear chromosome, and ROS can also promote the activation and transformation of tumoral cells. What has been reported is that the ROS level in tumors correlates with the degree of malignancy [13]. As ROS levels rise in hypoxia, malignant tumor cells become more aggressive and more likely to spread. Chronic and ongoing oxidative stress induces epithelial-mesenchymal transition (EMT) and migration [14]. It is evident that oxidative stress participates as a vital part in tumorigenesis and progression. At present, there is no report on predicting the development of breast cancer patients by building oxidative stress-related lncRNA prediction signals.

With this study, lncRNAs which are oxidative stress-related were screened by generating a lncRNA coexpression network and genes which are oxidative stress-related. Furthermore, using Lasso as well as Cox regression, the following seven lncRNAs which are oxidative stress-related with good prognosis were obtained: DLG5-AS1, LINC01235, SEMA3B-AS1, LINC00987, ST7-AS1, MAPT-AS1, and LINC01871. These seven lncRNAs which are oxidative stress-related may be targeting markers of potential clinical therapy and development for the BC patients. We also found mRNAs (MRPS34, HSPB1, GFER, NTHL1, UCN, F3, CDK5, GDF15, S100B, EGFR, STAT1, CALR, IL18, and IDO1) coexpressed significantly with the lncRNAs mentioned.

Five lncRNAs associated with oxidative stress (LINC01235, SEMA3B-AS1, LINC00987, ST7-AS1, and MAPT-AS1) have been reported to be linked to cancer. (1) Functional loss experiments suggest that upregulated LINC01235 promotes gastric cancer cell metastasis through EMT and may be a valuable prognostic biomarker and treating target for metastatic gastric cancer [15]. (2) Overexpression of SEMA3B-AS1 inhibits gastric cancer cell proliferation and invasion in vitro. Sema3b-as1 can be used as a tumor suppressor and as a clinical therapy target for antitumor therapy [16]. (3) Silencing LINC00987 inhibits proliferation and invasion of osteosarcoma cells by regulating FNBP1 expression through cavernous Mir-376A-5p [17]. (4) ST7-AS1 promotes the lung adenocarcinoma cells malignancy by regulating Mir-181B-5p/KPNA4 axis. Therefore, aiming at ST7-AS1 and KPNA4 or upregulation of Mir-181B-5p may be beneficial for the treating lung adenocarcinoma [18]. (5) MAPT-AS1 has been identified as a solid prognostic marker of renal clear cell carcinoma (ccRCC), inhibiting the invasion and proliferation of ccRCC [19]. And its upregulation were associated with positive survival in breast cancer patients [20].

One of the lncRNAs associated with oxidative stress, LINC01871, may serve as a marker of BC prognosis, but has not been studied in depth for the pathogenesis of BC [21]. Another lncRNA, DLG5-AS1, has not been studied for its prognostic significance in cancer. As a result, more research is needed to determine how this lncRNA affects the development of patients with BC via oxidative stress.

The development of BC was significantly predicted based on the characteristics of seven lncRNAs associated with oxidative stress. Consistent with previous studies, the OS of the low-risk group was higher. These results suggest that risk score features have some potential in prediction of survival condition. Univariate and multivariate Cox analysis results indicated that this trait might be used as an independent prognostic predictor. The model demonstrated superior distinction and accuracy based on the *c*-index, calibration curve, ROC curve, and internal validation data, indicating that it can be used as a possible predictive tool.

Subsequent GSEA results showed that macrophages scored higher in the group with high-risk. The results indicate that tumor-associated macrophages (TAMs) are key cells promoting tumor in tumoral microenvironment. Preclinical TAM stimulates progression of breast tumor, including tumor cell growth and metastasis. In BC models, TAMs also causes resistance to a number of therapies. The previous work found that oxidative stress signalling has a role in BC cell proliferation and migration. Initially, important components of oxidative stress signalling were discovered to substantially correlate with clinical and pathological characteristics of BC. These connections were not independent of TNM staging or clinical subtype, implying that oxidative stress activation is a common feature associated with BC development. Internal identification proved that the predicting signature has positive predictive performance. PCA suggested that seven lncRNAs associated with oxidative stress could be differentiated according to the oxidative stress condition of the patients.

The results of GSEA implied that macrophages scored higher in the high-risk group. It was revealed that tumor-associated macrophages (TAMs) are key cells promoting tumor in tumoral microenvironment. Preclinical TAMs stimulate progression of breast tumor, including tumor cell growth and metastasis. TAMs also attributed to resistance to a series of treatment in BC models [22].

Our study also showed that patients high ranked may be sensitive to and resistant to demethotrexate against TNF, CD80, CD276, SF4, and NRP1 immunotherapy and conventional chemotherapy drugs including AZD6482, bicaluamide, AKT inhibitor VIII, BMS.708163, imatinib, lapatinib, pazolparib, and toxic carotene. This suggests that the group of patients with high risk may alleviate the disease from the combination of immunotherapy and chemotherapy, providing the basis for precise, individualized treatment of BC patients.

However, there are some limitations to our study. First, external validation of data from other databases is required to test the suitability of the predictive signatures. Secondly, the mechanism of lncRNA oxidative stress in BC needs further experimental verification.

## 5. Conclusions

In conclusion, lncRNAs with oxidative stress features can independently predict BC prognosis, providing support for the underlying mechanism of oxidative stress of lncRNAs and their response to clinical treatment therapy within BC; however, more research is needed.

## Figures and Tables

**Table 1:** Multivariate Cox analyzation towards the lncRNAs on the basis of TCGA COAD data.

lncRNA	Coefficient	HR	95% CI of HR
DLG5-AS1	-0.651	0.521	0.307-0.887
LINC01235	0.400	1.492	1.184-1.881
SEMA3B-AS1	-0.245	0.783	0.563-1.088
LINC00987	-0.684	0.504	0.303-0.841
ST7-AS1	-1.181	0.307	0.139-0.675
MAPT-AS1	-0.782	0.458	0.281-0.744
LINC01871	-0.714	0.490	0.332-0.721

**Table 2:** Risk scores as well as clinical characteristics regarding BC through analysis of multivariate Cox regression.

Variable	HR	HR95L	HR95H	*p* value
Age	1.041	1.025	1.056	1.55*E*-07
Gender	0.572	0.079	4.168	0.582
Stage	1.770	1.057	2.963	0.030
T	0.962	0.712	1.299	0.802
M	1.420	0.616	3.273	0.411
N	1.191	0.892	1.591	0.235
Risk score	1.068	1.050	1.087	3.91*E*-14

**Table 3:** Clinical impacts of the risk score characteristics (as identified by the TCGA-COAD data).

Clinical	*n*	Mean	SD	*t*	*p*
Risk score					
Age					
>65	222	2.064	2.442	1.378	0.169
≤65	634	1.805	2.304		
Gender					
Female	845	1.878	2.354	1.322	0.212
Male	11	1.465	1.001		
Stage					
I-II	655	1.708	2.169	-3.282	0.001
III-IV	201	2.408	2.773		
T					
T1-2	734	1.794	2.21	-1.938	0.055
T3-4	122	2.342	2.987		
M					
M0	840	1.851	2.323	-1.504	0.153
M1	16	3.014	3.076		
N					
N0	420	1.661	2.087	-2.605	0.009
N1-3	436	2.076	2.55		

**Table 4:** The different clinical features of patients across separate cohorts.

Variables	Entire TCGA dataset (*n* = 856)	Validation cohort	
		Training cohort (*n* = 427)	Testing cohort (*n* = 429)
Age (%)			
≤65	634 (74.1)	321 (75.2)	313 (73.0)
>65	222 (26.9)	106 (24.8)	116 (27.0)
Gender (%)			
Female	845 (98.7)	419 (98.1)	426 (99.3)
Male	11 (1.3)	8 (1.9)	3 (0.7)
Stage (%)			
I + II	655 (76.5)	324 (75.9)	331 (77.2)
III + IV	201 (23.5)	103 (24.1)	98 (22.8)
T (%)			
T1+T2	734 (85.7)	369 (86.4)	365 (85.1)
T3+T4	122 (14.3)	58 (13.6)	64 (14.9)
M (%)			
M0	840 (98.1)	420 (98.4)	420 (97.9)
M1	16 (1.9)	7 (1.6)	9 (2.1)
N (%)			
N0	420 (49.1)	210 (49.2)	2109 (49.0)
N1+N2+N3	436 (50.9)	217 (50.8)	219 (51.0)

M: metastasis; N: lymph node; T: tumor.

## Data Availability

The dataset used in this paper are available from the corresponding author upon request.
